# The assessment of complementary and alternative medicine use in acne vulgaris patients in Turkey: A cross-sectional study: Erratum

**DOI:** 10.1097/MD.0000000000042099

**Published:** 2025-04-04

**Authors:** Bahar Ürün Ünal, Abdullah Demirbaş, Kamile Marakoğlu, Burcu Gök Erdoğan

The article “The assessment of complementary and alternative medicine use in acne vulgaris patients in Turkey: A cross-sectional study”,^[1]^ which appears in Volume 103, Issue 37 of *Medicine*, was originally published with incorrect data. The study date range was updated from “February and October 2020” to “April and October 2020” in the published version. The change appears in the abstract and in the first paragraph of the “Material and methods” section.
